# Bufalin attenuates the stage and metastatic potential of hepatocellular carcinoma in nude mice

**DOI:** 10.1186/1479-5876-12-57

**Published:** 2014-02-28

**Authors:** Zhou-Ji Zhang, Yun-Ke Yang, Wei-Zhong Wu

**Affiliations:** 1 Department of Traditional Chinese Medicine, Zhongshan Hospital, Fudan University, 180 Fenglin Rd, Shanghai 200032, China; 2 Key Laboratory of Carcinogenesis and Cancer Invasion, Ministry of Education, Liver Cancer Institute and Zhongshan Hospital, Fudan University, 180 Fenglin Rd, Shanghai 200032, China

**Keywords:** Hepatocellular carcinoma, Pulmonary metastases, Orthotopic transplantation tumor models, AKT/GSK3β/β-catenin/E-cadherin signaling pathways

## Abstract

**Background:**

Advanced hepatocellular carcinoma (HCC) patients undergo significant tumor growth and metastasis. Here, we investigated bufalin for treating HCC, which exhibits anti-tumor activities in many tumor cell lines.

**Method:**

In our experiment, HCCLM3-R cells were injected into nude mice to form subcutaneous human HCC tumors that were implanted into the liver to establish orthotopic transplantation tumor models. Bufalin was injected intraperitoneally at 1 or 1.5 mg/kg. LY294002 (100 mg/kg), a potent inhibitor of Akt which reduced the levels of pAkt in HCCLM3 cell lines, was injected intraperitoneally into one group thrice weekly. The control was injected with an equal volume of saline. Morphological alterations were evaluated in the liver and lung by stereomicroscopy, the apoptotic rate was measured by TUNEL staining, and expression of AKT/GSK3β/β-catenin/E-cadherin signaling pathway-related proteins was detected by immunohistochemistry (IHC) and western blot analysis.

**Results:**

These results suggested that the sizes and qualities of orthotopic transplanted tumors as well as pulmonary metastasis decreased markedly at the highest bufalin dose compared with that in the control. Orthotopic transplanted tumor tissues were necrotic in bufalin-treated groups and the apoptotic cell number was markedly higher at the highest bufalin dose compared with that in the control. Certain changes of expression of AKT/GSK3β/β-catenin/E-cadherin signaling pathway-related proteins were in tumor tissues, which were related to the bufalin dose. Similar results were observed in the LY294002-treated group.

**Conclusion:**

Based on the above, one can draw conclusions that bufalin has significant anti-tumor activities and reduces the metastatic potential in an orthotopic transplantation tumor model of human HCC. Inhibition of AKT/GSK3β/β-catenin/E-cadherin signaling pathways by bufalin may show therapeutic effects in advanced HCC patients.

## Background

Hepatocellular carcinoma (HCC) is the most common primary liver tumor and the third highest cause of cancer-related deaths worldwide (696,000 deaths, 9.2%) [1]. The high mortality rate is related to poor early diagnosis because of a lack of observable symptoms, as well as the aggressiveness of the disease and limited therapeutic options. Thus, most HCC patients are diagnosed with advanced disease and generally have a poor prognosis with median survival times of about 6–8 months [2]. However, patients that undergo surgical resection or liver transplantation at an early stage of HCC have 5-year overall survival rates of 30–40% [3] and 61–89%, respectively [4]. The short survival time of HCC patients is associated with tumor initiation, progression, and metastasis. Recently, the phosphoinositide 3-kinase/protein kinase B (PI3K/Akt) signaling pathway has been found to play an essential role in cancer cell proliferation, survival, metabolism, motility, and invasion, thereby facilitating the formation of clinical metastases [5]. The pro-survival activity of the PI3K/Akt signaling pathway has been investigated in great detail in human physiology and disease. Previous studies have demonstrated that this signaling axis is actively engaged in metastatic cancer cells [6]. AKT is also instrumental in angiogenesis and epithelial-to-mesenchymal transition during tumorigenesis [7]. Nude mouse experiments have shown that suppression of the PI3K/AKT signaling pathway can inhibit tumor growth, reduce angiogenesis, and improve the tumor microenvironment [8].

Bufalin is the major bioactive component of venenum bufonis with antitumor activity, which is a traditional Chinese medicine obtained from the skin and parotid venom glands of toads [9]. In previous studies, bufalin has been demonstrated to induce apoptosis in gastric cancer MGC803 cells and oral cancer CAL 27 cells by inhibition of the AKT signaling pathway [10,11]. Our previous in vitro studies have shown that the mechanisms underlying the antitumor effects of bufalin in hepatoma cells appear to be mediated by AKT/GSK3β/β-catenin/E-cadherin signaling pathways [12].

The present study used an orthotopic human HCC model with high metastatic potential in nude mice [13]. We observed the effects of bufalin on inhibition of HCC growth and metastatic potential and explored the mechanism of the AKT signaling pathway in the nude mouse model.

## Methods

### Animals

Male athymic BALB/c nu/nu mice (18–20 g, 5 weeks old) were obtained from the Shanghai Institute of Materia Medica, Chinese Academy of Science. All mice were handled according to the recommendations of the National Institutes of Health Guidelines for Care and Use of Laboratory Animals. The experimental protocol was approved by the Shanghai Medical Experimental Animal Care Committee.

### HCC cell line

Stable red fluorescent protein-expressing HCCLM3 (HCCLM3-R) cells infected with a lentivirus containing full-length cDNA of red fluorescent protein were used in this study [13].The HCCLM3-R cells were maintained at 37°C with 5% CO_2_ in Dulbecco’s modified Eagle medium (DMEM) supplemented with 10% heat-inactivated fetal bovine serum (FBS).

### Treatments

Bufalin (purity >98%) was purchased from Shanghai Tauto Biotech Co., Ltd. (Shanghai, China) and dissolved in ethanol at a concentration of 0.2 g/L. LY294002 was purchased from Cell Signaling Technology (Beverly, MA, USA) and diluted in phosphate buffered saline (PBS) at a concentration of 13 g/L. Reagents were sterilized by passing through a 0.22 μm filter and stored at 4°C.

### Metastatic orthotopic tumor model in nude mice

A metastatic model of human HCC in nude mice using HCCLM3-R cells was employed for this study [13]. Briefly, HCCLM3-R cells (5 × 10^6^) were injected subcutaneously into the upper left flank region of nude mice. When the subcutaneous tumors reached approximately 1 cm in length (approximately 4 weeks after injection), they were removed, minced into small pieces of equal volume (1.5–2 × 2 × 2 mm^3^), and transplanted into the livers of 24 nude mice. The animals were randomly divided into four groups (n = 6). Based on the literature [14,15] and the results of our preliminary experiments, we used physiological saline to dilute the bufalin and LY294002. Bufalin was intraperitoneally injected into two groups of mice at doses of 1 mg/kg and 1.5 mg/kg from day 8 to 38, and LY294002 was intraperitoneally into one group of mice at a dose of 100 mg/kg thrice weekly as a positive control. The control group was injected with an equal volume of saline (0.15 ml) from day 8 to 38. All mice were sacrificed and weighed on day 39.

### Analysis of tumor necrosis and apoptosis

Paraffin-embedded sections were prepared for hematoxylin and eosin (H&E) staining. Tumor tissue necrosis was determined by comparing the surface of necrotic areas with that of the whole tumor. Apoptosis was determined using a terminal transferase dUTP nick end labeling (TUNEL) assay kit according to the manufacturer’s protocol. A positive sample contained 25 positively stained cells in every 100 tumor cells calculated randomly from five high magnification (400×) fields for each specimen.

### Detection of metastasis by fluorescence microscope and H&E staining

Tumors were excised, weighted, and the largest (*a*) and smallest (*b*) diameters were measured to calculate the tumor volume (*V*) with the formula: *V* = *ab*^2^/2 [16]. And the inhibition rates of tumors with the formula: inhibition rates (%) = (NS tumor weight (g)-medication tumor weight (g))/NS tumor weight (g) × 100%). The lungs and mesenteries were also excised, red fluorescent protein-positive metastatic foci were analyzed by fluorescence microscopy, and the integrated absorbance was quantitated by Image-Pro Plus software as described previously [17]. Then, orthotopic tumors and lungs were fixed with 10% buffered formalin and embedded in paraffin. Serial sections of pulmonary metastatic nodules and mesenteries were cut at 5 μm intervals and stained with H&E [12].

### Immunohistochemical staining for phosphorylated (p)-AKT, AKT, p-GSK3β, GSK3β, β-catenin, E-cadherin, MMP-2, and MMP-9

To assess the distribution of p-AKT and AKT(purchased from Cell Signaling Technology, Inc. (Beverly, MA, USA)), p-GSK3β, GSK3β, β-catenin, E-cadherin, MMP-2 and MMP-9 (purchased from Epitomics, Inc. (Burlingame, CA, USA)), we stained sections with rabbit anti-p-Akt (1:250), anti-Akt (1:100), anti-pGSK3β (1:100), anti-GSK3β (1:200), anti-β-catenin (1:200), anti-E-cadherin (1:200), anti-MMP-2 (1:150), and anti-MMP-9 (1:100) antibodies. A rabbit anti-rat fluorescein isothiocyanate-conjugated antibody (1:100) was applied as the secondary antibody. All sections were independently assessed by two board-certified pathologists who were blinded to the experiment. For negative controls, primary antibodies were replaced with PBS. Positive samples contained 25 positively stained cells in every 100 tumor cells calculated randomly from five high magnification (400×) fields for each specimen. After immunohistochemistry (IHC), morphometrical analysis was conducted with the Imageproplus color image analysis system. One-hundred positive cells in each positive sample were chosen randomly under 200× and 400× magnification. Then, the average optical density (AOD) and integral optical density (IOD) of positive granules were detected as relative amounts of p-AKT, AKT, p-GSK3β, GSK3β, β-catenin, E-cadherin, MMP-2, and MMP-9 in cells. Positive cells in HCC xenografts were calculated as follows: AOD = IOD/total sample area [18].

IHC staining was also scored as follows: IHC 0 (completely negative), IHC 1+ (faint membranous or cytoplasmic positivity), IHC 2+ (moderate, smooth membranous or cytoplasmic positivity), IHC 3+ (strong, more than 30% tumor cells with circumferential membranous or intense, granular cytoplasmic positivity ) in more than 10% of tumor cells. Scoring was performed by two pathologists [19].

### Western blotting analysis

Fresh tumor tissues in RIPA lysis buffer containing 1 ug/ml PMSF were manually homogenized on ice using a glass homogenizer, then centrifuged at 10000 g for 10 min to remove celluar and nuclear debris. The protein concentrations were determined with the BCA protein assay, and equal amounts of protein were subjected to 12% sodium dodecyl sulfate polyacrylamide gel electrophoresis (SDS-PAGE). After electrophoresis, proteins were transferred onto polyvinylidene difluoride membranes (PVDF). The membranes were blocked for 1 hour at room temperature in 5% nonfat dry milk in Phosphate Buffered Saline with Tween-20 (PBST), followed by overnight incubation at 4°C with primary antibodies. The membranes were washed and then incubated with a peroxidase conjugated secondary antibody for 1 hour at room temperature. Blots were detected through chemiluminescence substrate (ECL plus) and acquired by digital images.Primary antibodies used include rabbit anti-pAkt (1:100), anti-Akt (1:1000), anti-pGSK3β (1:10000),anti-GSK3β (1:5000), anti-β-catenin (1:5000), anti-E-cadherin (1:5000), anti-MMP-2 (1:1000), anti-MMP-2 (1:1000) and GAPDH(1:1000).

### Statistical analyses

Statistical analyses were performed with IBM SPSS Statistics 19 for Windows. Quantitative variables were analyzed by analysis of variance and expressed as the mean ± SD. Results were considered statistically significant at P < 0.05.

## Results and discussion

### Bufalin inhibits tumor growth but has no effect on nude mouse survival

After 4 weeks of treatment, high-dose bufalin-treated mice had smaller tumor volumes compared with those of mice in the control group (712.88 ± 212.12 mm^3^ vs. 1379.75 ± 741.48 mm^3^, P < 0.05) and lower tumor weights compared with those of mice in the control group (1.10 ± 0.23 g vs. 1.93 ± 0.45 g, P < 0.01). However, there were no significant differences between low-dose bufalin-treated mice and the control group in terms of their tumor volumes (1174.69 ± 692.42 mm^3^ vs. 1379.75 ± 741.48 mm^3^, P > 0.05 ) and weights (1.65 ± 0.60 g vs. 1.93 ± 0.45 g, P > 0.05).

**Figure 1:**
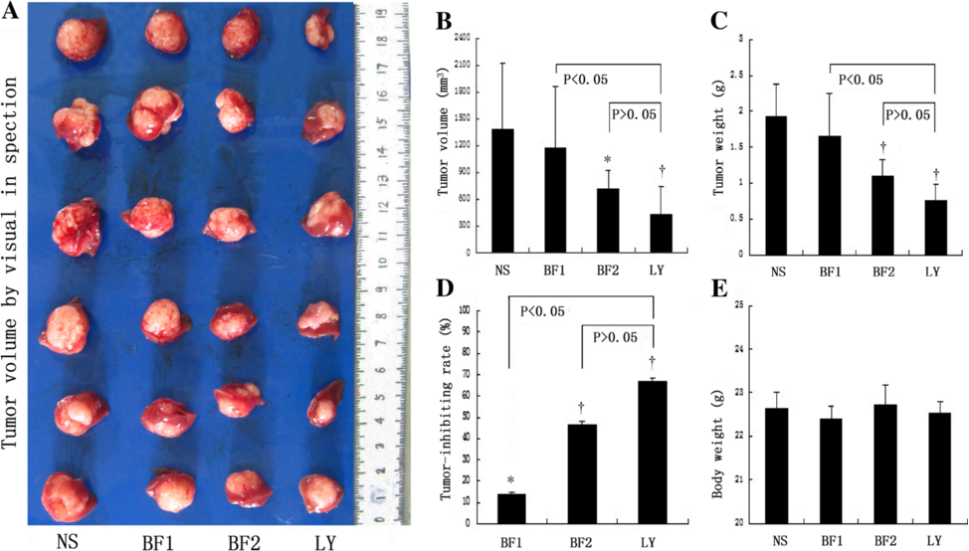
**Bufalin inhibits tumor growth but has no effect on nude mouse survival. (A)** Tumor volumes of control (NS), low-dose bufalin-treated (BF1), high-dose bufalin-treated (BF2), and LY294002-treated (LY) groups by visual inspection. **(B)** Quantitation of tumor volumes in control (NS), low-dose bufalin-treated (BF1), high-dose bufalin-treated (BF2), and LY294002-treated (LY) groups. **(C)** Quantitation of tumor weights in control (NS), low-dose bufalin-treated (BF1), high-dose bufalin-treated (BF2), and LY294002-treated (LY) groups. **(D)** Tumor inhibition rates of control (NS), low-dose bufalin-treated (BF1), high-dose bufalin-treated (BF2), and LY294002-treated (LY) groups. **(E)** Nude mouse body weights of control (NS), low-dose bufalin-treated (BF1), high-dose bufalin-treated (BF2), and LY294002-treated (LY) groups. All data represent the mean ± SD (n = 6). *P < 0.05 vs. control (NS); †P < 0.01 vs. control (NS).

LY294002-treated mice had the smallest tumor volumes (438.35 ± 303.37 mm^3^) and lowest tumor weights (0.76 ± 0.23 g), which were not obviously different from those in the high-dose bufalin-treated group (P > 0.05) (Figure 1A–C). The inhibition rates of tumors were 13.51 ± 1.35% in low-dose bufalin-treated mice, 46.27 ± 2.06% in high-dose bufalin-treated mice, and 66.83 ± 1.40% in LY294002-treated mice (Figure 1D). On the other hand, pre- and-post-treatment variation of nude mouse body weights among the four groups showed no significant differences. Also during the process, appetites and behaviors of these four groups nude mouse were much the same. In addition, there were no treatment-related deaths (Figure 1E). Collectively, bufalin inhibited tumor growth in nude mice in a dose-dependent manner but had no effect on nude mouse survival.

### Orthotopic transplanted tumor tissues undergo necrosis and apoptosis induced by bufalin treatment

**Figure 2:**
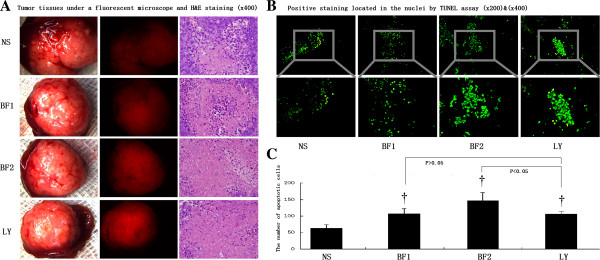
**Orthotopic transplanted tumor tissues undergo necrosis and apoptosis induced by bufalin treatment. (A)** Detection of transplanted tumors in control (NS), low-dose bufalin-treated (BF1), high-dose bufalin-treated (BF2), and LY294002-treated (LY) groups by fluorescence microscopy and necrotic tumor tissues by H&E staining (×400). **(B)** Positive TUNEL staining in the nuclei of control (NS), low-dose bufalin-treated (BF1), high-dose bufalin-treated (BF2), and LY294002-treated (LY) groups (×200 and ×400). **(C)** TUNEL staining showed different apoptotic rates in control (NS), low-dose bufalin-treated (BF1), high-dose bufalin-treated (BF2), and LY294002-treated (LY) groups. All data represent the mean ± SD (n = 6). *P < 0.05 vs. control (NS); †P < 0.01 vs. control (NS).

The in situ transplanted tumors exhibited relatively regular round or oval shapes with complete or incomplete capsules. Compared with the control, mice treated with bufalin had a significantly smaller tumor volume. H&E staining revealed that the nuclei of the liver cancer cells were large with an oval shape and contained more chromatin. Binucleated cells were large and clearly visible with more free ribosomes and more pathological karyokinesis. Severe necrosis was mainly observed in high-dose bufalin-treated and LY294002-treated groups, whereas there was moderate necrosis in the low-dose bufalin-treated group and mild necrosis in the control group. Tumor cells with less cytoplasm were observed in high-dose bufalin-treated and LY294002-treated groups compared with those in the control group, including apoptotic signs such as pyknotic nuclei, chromatin condensation around the edges, and clefts in the nuclei (Figure 2A). Positive TUNEL staining was located in the nuclei (Figure 2B). The numbers of apoptotic cells in control, low-dose bufalin-treated, high-dose bufalin-treated, and LY294002-treated groups were 63.20 ± 10.33, 107.80 ± 14.20, 146.80 ± 14.20, and 106.20 ± 7.12 per 400× magnification field, respectively (Figure 2C). Therefore, orthotopic transplanted tumor tissues undergo necrosis and apoptosis induced by bufalin treatment.

### Bufalin inhibits pulmonary metastases

**Figure 3:**
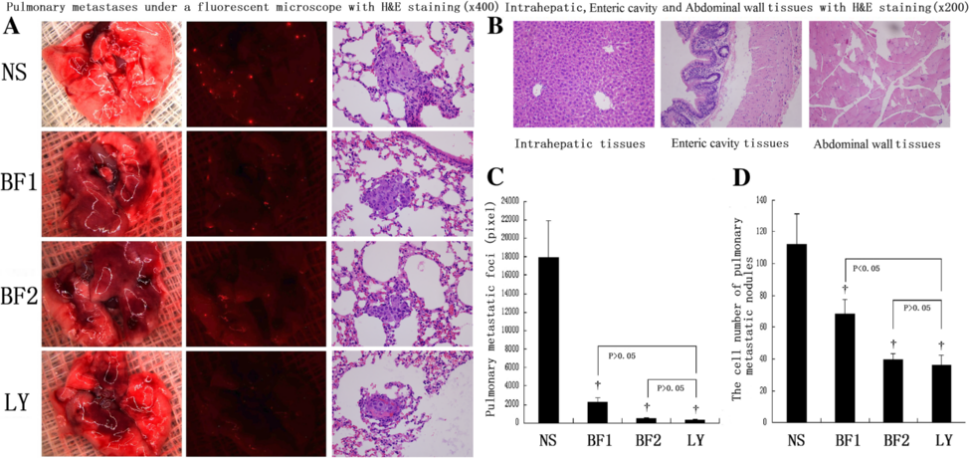
**Bufalin inhibits pulmonary metastases. (A)** Detection of pulmonary metastases in control (NS), low-dose bufalin-treated (BF1), high-dose bufalin-treated (BF2), and LY294002-treated (LY) groups by fluorescence microscopy and necrotic tumor tissues by H&E staining (×400). **(B)** Detection of intrahepatic, enteric cavity, and abdominal wall tissues by H&E staining (×200). **(C)** The pixel numbers of pulmonary metastatic foci in control (NS), low-dose bufalin-treated (BF1), high-dose bufalin-treated (BF2), and LY294002-treated (LY) groups. **(D)** The cell numbers of pulmonary metastatic nodules in control (NS), low-dose bufalin-treated (BF1), high-dose bufalin-treated (BF2), and LY294002-treated (LY) groups. All data represent the mean ± SD (n = 6). †P < 0.01 vs. control (NS).

Pulmonary metastases were also significantly inhibited by bufalin in the HCCLM3-R cell model. Bioluminescence showed that the pixel numbers of pulmonary metastatic foci in high-dose and low-dose bufalin-treated mice were significantly less than those in control mice (512.00 ± 95.83 and 2217.00 ± 475.07 vs. 17937.33 ± 3997.06 per lung, P < 0.01, respectively). In H&E staining, a decrease in the cell number of pulmonary metastatic nodules was found in high-dose and low-dose bufalin-treated groups compared with that in the control (39.67 ± 3.72 and 68.17 ± 9.47 vs. 112.17 ± 19.05 per lung, P < 0.01, respectively). A similar trend toward reduction of the pixel number of pulmonary metastatic foci (356.50 ± 83.29 vs. 17937.33 ± 3997.06 per lung, P < 0.01) and cell number of pulmonary metastatic nodules (36.33 ± 5.99 per lung, P < 0.01) was also observed in LY294002-treated mice with HCCLM3-R xenografts compared with those in control mice (Figure 3A, C and D). After exploratory laparotomy and observing the histological characteristics of the samples, no intrahepatic dissemination, peritoneal dissemination, or abdominal lymph node metastasis were found in all four groups of the HCCLM3-R xenograft model (Figure 3B).

Taken together, analysis of serial lung sections indicated significant inhibition of pulmonary metastases in bufalin-treated mice.

### Bufalin-treated tumors exhibit changes consistent with the AKT/GSK3β/β-catenin/E-cadherin signaling pathway in nude mice

On day 39 after treatments, control, low-dose bufalin-treated, high-dose bufalin-treated, and LY294002-treated hepatomas were subjected to IHC. First, treatment of HCCLM3-R xenografts in nude mice with bufalin inhibited Akt phosphorylation. High-dose and low-dose bufalin-treated groups had lower average Akt phosphorylation levels compared with those in the control group. The LY294002-treated group had the lowest Akt phosphorylation levels. In addition, bufalin treatment enhanced total GSK3β expression and inhibited GSK3β serine 9 phosphorylation in a dose-dependent manner. Total GSK3β expression of tumors was IHC 1+ in low-dose bufalin-treated mice, IHC 2+ in high-dose bufalin-treated mice and IHC 0 in the control. Furthermore, GSK3β serine 9 phosphorylation in control, low-dose bufalin-treated, and high-dose bufalin-treated groups was IHC 3+, IHC 2+, and IHC 1+, respectively. The reduction of p-Akt expression appeared without any change in the total amount of Akt (similar protein levels among the four groups). Moreover, expression of p-GSK3β was significantly down-regulated with the increase in the total amount of GSK3β by bufalin treatment. Both total GSK3β and p-GSK3β showed analogous expression in the LY294002-treated group (Additional file 1: Figure S1A and B).

Second, the highest levels of β-catenin at the cell membrane were observed in the control group, which decreased with bufalin treatment in low-dose and high-dose bufalin-treated groups. The LY294002-treated group had the lowest β-catenin protein level.

Third, untreated tumors showed typical membranous E-cadherin expression at cell-cell contacts, and E-cadherin-positive cells were localized in central tumor areas. IHC exhibited weak staining of E-cadherin in the control group. In contrast, bufalin-treated groups showed an overall increase of E-cadherin expression. The LY294002-treated group had the highest E-cadherin protein level (Additional file 1: Figure S1C).

Finally, MMP-2 protein levels were significantly downregulated and expression of MMP-9 protein also decreased in bufalin-treated groups compared with that in the control. High-dose bufalin-treated mice had lower MMP-2 and MMP-9 protein levels compared with those in the control and low-dose bufalin-treated mice. LY294002-treated mice had the lowest MMP-2 and MMP-9 protein levels (Additional file 1: Figure S1D).

In summary, changes in the expression of numerous important regulatory proteins (Additional file 1: Figure S1E) in the AKT/GSK3β/β-catenin/E-cadherin signaling pathway occurred in a dose-dependent manner by treatment with bufalin.

### Bufalin was able to modulate protein expressions consistent with AKT/GSK3β/β-catenin/E-cadherin signaling pathway by western blot analysis

**Figure 4:**
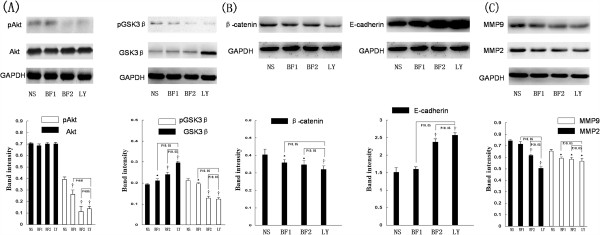
**Bufalin can modulate protein expressions consistent with AKT/GSK3β/β-catenin/E-cadherin signaling pathway by western blot analysis. (A)** Protein levels and quantitative analysis of pAKT, AKT, pGSK3β and GSK3β were analyzed by western blotting. **(B)** Protein levels and quantitative analysis of β-catenin and E-cadherin were analyzed by western blotting. **(C)** Protein levels and quantitative analysis of MMP2 and MMP9 were analyzed by western blotting. All data represent the mean ± SD (n = 6). *P < 0.05 vs. control (NS); †P < 0.01 vs. control (NS).

On day 39 after treatments, control, low-dose bufalin-treated, high-dose bufalin-treated, and LY294002-treated hepatomas were subjected to western blot analysis. Bufalin significantly downregulated the expression of pAKT in HCCLM3-R cells without affecting the total protein levels of AKT. Also, bufalin significantly suppressed the phosphorylation of GSK protein and increased GSK3β protein activation. Also, bufalin significantly suppressed β-catenin protein. Then we further investigated the downstream molecular actions of E-cadherin after the inhibitory β-catenin protein. We found that bufalin significantly increased E-cadherin expression in HCCLM3-R cells. The association between MMP expression and the invasive activity of various types of cancer provided by the loss of E-cadherin had been well documented. Bufalin can regulate the expression of MMP-9 and MMP-2 at the transcriptional level in hepatoma cells. Furthermore, bufalin inhibited the expression of pAKT, AKT, pGSK3β, GSK3β, β-catenin, E-cadherin MMP-2 and MMP-9 in HCCLM3-R in a dose-dependent manner. On the other hand, LY294002, a potent inhibitor of AKT, acted on the levels of pAKT, AKT, pGSK3β, GSK3β,β-catenin, E-cadherin MMP-2 and MMP-9 observed in mice with HCCLM3-R xenografts hepatomas display similar trends to bufalin-treated group (Figure 4).

In short, bufalin was able to modulate protein expressions consistent with AKT/GSK3β/β-catenin/E-cadherin signaling pathway in HCCLM3-R cells by western blot analysis

## Conclusions

HCC is the most common primary liver tumor and is defined as a high-mortality disease [1]. Metastasis begins with cell outgrowth from a primary tumor, followed by invasion and survival in stromal tissues, which is responsible for 90% of cancer patient deaths [20]. Therefore, it is particularly important to improve patient survival to control the tumor-initiating and metastatic properties of HCC.

Currently, there are no reports of the influence of bufalin on the stage and metastatic potential of HCC in vivo. Therefore, to study tumor growth and pulmonary metastases under exposure to bufalin, we established orthotopic transplantation tumor models of human HCC in nude mice using HCCLM3-R cells.

We found that doses of 1 and 1.5 mg/kg bufalin inhibited tumor growth, including reductions of the tumor volume and weight, and increased the tumor inhibition rate, which was more apparent at the highest dose of bufalin. In addition, bioluminescence showed clear dose-dependent inhibition of pulmonary metastasis in bufalin-treated mice. The number of pixels indicating pulmonary metastatic foci showed a significant decrease in the high-dose bufalin-treated group. Combined with our previous in vitro results concerning the effects of inhibiting hepatoma cell proliferation, migration, invasion, and adhesion using bufalin [12], we conclude that bufalin has the same anti-tumor effects in vitro and in vivo.

Our previous study also showed that bufalin inhibits expression of p-AKT in human hepatoma cells in a time-dependent manner [12]. Therefore, in this study, we investigated the effect of bufalin on AKT expression in vivo. Bufalin-treated groups had lower p-AKT levels than those in the control group, and the lowest level of p-AKT was observed in the high-dose bufalin-treated group. On the other hand, there were no obvious changes in total protein levels of AKT among the groups. Therefore, the change of p-AKT levels might be related to tumor growth and the pulmonary metastatic potential of HCC in the nude mouse model. Deregulation of AKT signaling is widely found in a variety of human cancers by elevation of Akt activity that is determined by p-AKT expression. Activated AKT plays critical roles in metastasis, including escape of the cells from the tumor environment, activation of cell proliferation, inhibition of apoptosis, and initiation of angiogenesis [21].

One of the major downstream effectors of Akt is GSK3β, a multifunctional serine/threonine kinase. GSK3β levels may be regulated by bufalin treatment, which requires further exploration. Our study showed that p-GSK3β expression was significantly down-regulated, while total GSK3β increased by bufalin treatment. Upon Akt activation, Akt inactivates GSK3β by initiating its phosphorylation at serine 9 and vice versa [22], which supports our results. GSK3β also regulates various cellular functions such as cell survival and cell-fate specification, and participates in numerous signaling pathways linked to diverse physiological processes and pathological conditions [23]. It is well known that GSK3β negatively regulates the classical Wnt signaling pathway by phosphorylation of β-catenin, and activation of GSK3β induces ubiquitin-dependent degradation of β-catenin [24]. Our previous in vitro study showing the loss of nuclear β-catenin accumulation by bufalin treatment indicates that it can modulate switching between membrane- and nuclear-associated β-catenin [12]. In this study, considering the relationship between GSK3β and β-catenin expression, we investigated β-catenin levels and found a significant decrease of β-catenin levels in bufalin-treated groups. Therefore, our in vitro and in vivo studies have confirmed a decrease of β-catenin levels by rapid proteolytic degradation after its phosphorylation by GSK3β, and an enhancement of activated GSK3β levels by bufalin treatment. Many studies have indicated that the hallmark of β-catenin signaling in normal and neoplastic tissues is nuclear translocation, and activated GSK3β decreases nuclear β-catenin accumulation and transcriptional activity, suggesting its potent inhibitory function in Wnt/β-catenin signaling [25].

β-Catenin is a key component of E-cadherin-mediated cell-cell adhesion that is predominantly associated with the cytoplasmic domain of E-cadherin at neighboring cell junctions in normal epithelial cells [26]. Immunohistochemical staining exhibited no staining of E-cadherin in the control group, whereas bufalin-treated groups showed weak increase of E-cadherin expression. This phenomenon is related to the proposed mechanism of the AKT signaling pathway involving β-catenin. Our western blot analysis indicated noticeable changes of tumor β-catenin levels in the HCCLM3-R cell line and the reduction of β-catenin expression significantly increases E-cadherin expression.

Therefore, our results suggest that bufalin inhibits tumor growth and reduces pulmonary metastases in orthotopic transplantation tumor models of human HCC in nude mice, and the underlying mechanism is partly mediated by AKT/GSK3β/β-catenin/E-cadherin signaling pathways.

Further investigation of the pathological changes of tumor tissues in bufalin-treated groups by H&E staining and TUNEL assays showed different degrees of necrosis and apoptosis. These results indicate a close relationship of the actions of bufalin in the inhibition of HCC growth and enhancement of necrosis and apoptosis in orthotopic-transplanted tumor tissue, which are related to regulation of the PI3K/Akt signaling pathway. Therefore, our results are consistent with those of previous studies showing that bufalin induces apoptosis in gastric cancer MGC803 cells and oral cancer CAL 27 cells by inhibition of the PI3K/AKT signaling pathway [10,11].

Our in vivo results revealed that bufalin was able to decrease MMP-2 and MMP-9 expression levels in a dose-dependent manner, whereas our previous in vitro experiments show that bufalin is able to decrease MMP-2 and MMP-9 expressions in hepatoma cell lines [12]. MMP-2 and MMP-9 are zinc-dependent endopeptidases that play critical roles in cancer progression and metastasis [27]. Previous observations show that upregulation of E-cadherin and a concomitant reduction in MMP-2/MMP-9 levels might negatively regulate cell proliferation, invasiveness, and adhesion of K1 papillary thyroid cancer cells and pancreatic cancer cell lines MIAPaCa-2 and BxPC-3 [28,29]. However, other studies show that inhibition of the β-catenin pathway might play a central role in anticancer activities through modulation of MMPs [30]. Therefore, there has been intense investigation to reduce MMP levels through inhibition of the β-catenin pathway or upregulation of E-cadherin expression levels. In future studies, we will clarify the mechanism of MMP-2 and MMP-9 downregulation by bufalin treatment.

Our study also revealed no differences of nude mouse survival, body weights, appetites, or behaviors among bufalin-treated groups and the control, indicating the safety and efficacy of bufalin treatment. Based on our results, the next step is large-scale clinical studies of bufalin treatment.

In summary, our results suggest that bufalin exhibits multiple antitumor effects in orthotopic transplantation tumor models of human HCC in nude mice, such as inhibition of tumor growth, leading to tumor tissue necrosis and apoptosis as well as a reduction of pulmonary metastasis. The mechanisms underlying bufalin actions appear to be mediated by AKT/GSK3β/β-catenin/E-cadherin signaling pathways. Bufalin is a promising anti-HCC agent, and further studies should be performed in advanced HCC patients.

## Abbreviations

HCC: Hepatocellular carcinoma; IHC: Immunohistochemistry; PI3K/Akt: Phosphoinositide 3-kinase/protein kinase B; HCCLM3-R: Stable red fluorescent protein-expressing HCCLM3; DMEM: Dulbecco’s modified Eagle medium; FBS: Fetal bovine serum; PBS: Phosphate buffered saline; H&E staining: Hematoxylin and eosin; TUNEL: Terminal transferase dUTP nick end labeling; AOD: Average optical density; IOD: Integral optical density; SDS-PAGE: Sodium dodecyl sulfate polyacrylamide gel electrophoresis; PVDF: Polyvinylidene difluoride membranes; PBST: Phosphate buffered saline with tween-20; NS: Control; BF1: Low-dose bufalin-treated; BF2: High-dose bufalin-treated; LY: LY294002-treated.

## Competing interests

The authors declare that they have no competing interests.

## Authors’ contributions

YKY and WZW conceived the project. ZJZ organized the study, analyzed the effects of bufalin on attenuating the stage and metastatic potential of hepatocellular carcinoma in nude mice, and helped to prepare the manuscript. ZJZ and YKY performed the statistical and cell signaling pathway analyses. WZW and YKY contributed to the interpretation of the results and helped to write the manuscript. All the authors read and approved the final manuscript.

## Supplementary Material

Additional file 1: Figure S1 Bufalin-treated tumors exhibit changes consistent with AKT/GSK3β/β-catenin/E-cadherin signaling pathways in nude mice. **(A)** Immunohistochemical staining for tumor p-AKT and AKT protein expressions (×200, ×400, ×1000). **(B)** Immunohistochemical staining for tumor p-GSK3β and GSK3β protein expressions (×200, ×400, ×1000). **(C)** Immunohistochemical staining for tumor β-catenin and E-cadherin protein expressions (×200, ×400, ×1000). **(D)** Immunohistochemical staining for tumor MMP-2 and MMP-9 protein expressions (×200, ×400, ×1000). **(E)** Quantitative analysis of expression of p-AKT, AKT, p-GSK3β, GSK3β, β-catenin, E-cadherin, MMP-2 and MMP-9 protein. All data represent the mean ± SD (n = 6). †P < 0.01 vs. control (NS). Click here for file
