# Supplementary figures and images for: Bufalin attenuates the stage and metastatic potential of hepatocellular carcinoma in nude mice
Source: J Transl Med. 2014 Feb 28;12:57. doi: 10.1186/1479-5876-12-57 (PMC4015709; doi:10.1186/1479-5876-12-57)

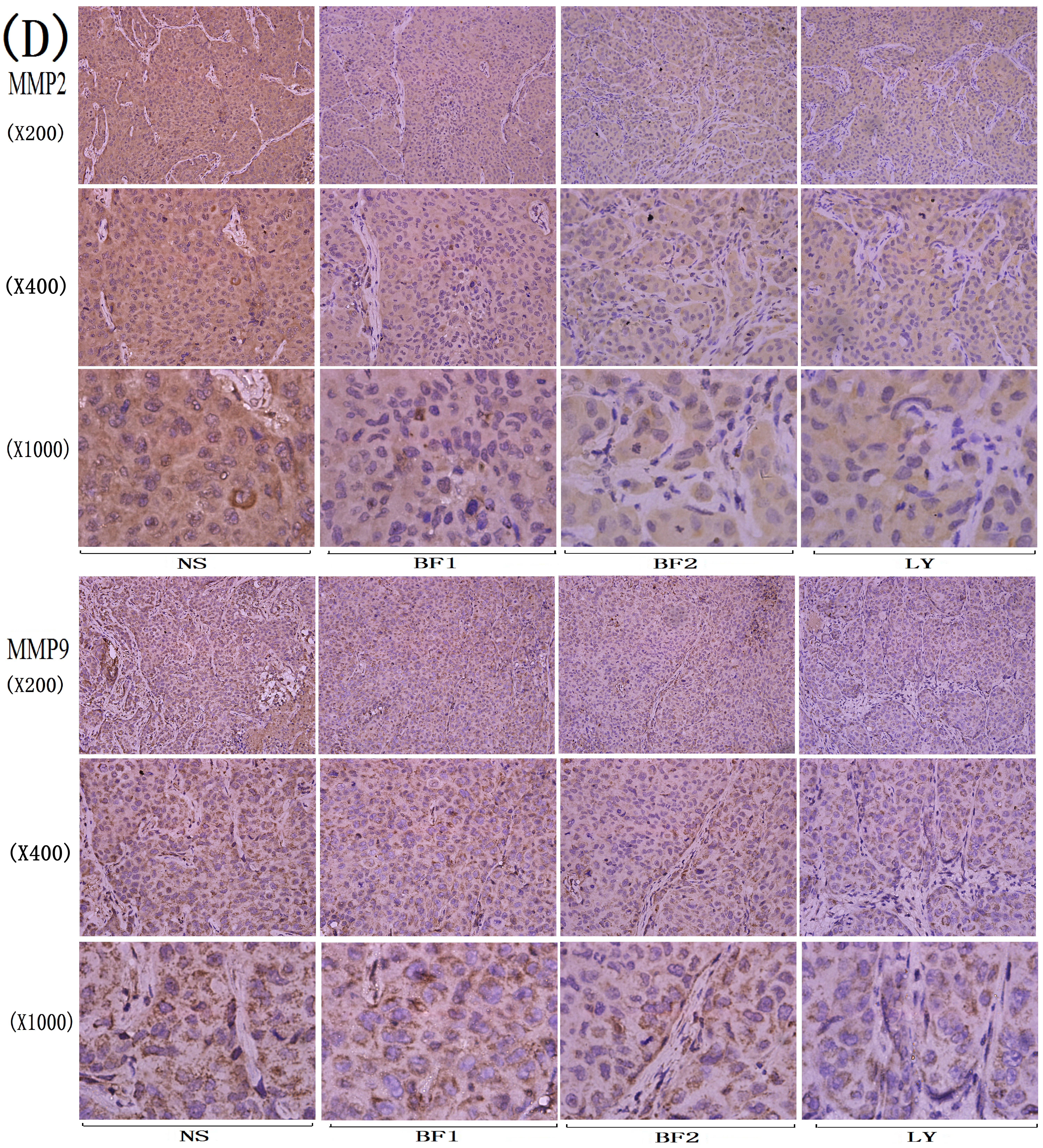

Supplement: Additional file 1: Figure S1 — Bufalin-treated tumors exhibit changes consistent with AKT/GSK3β/β-catenin/E-cadherin signaling pathways in nude mice. (A) Immunohistochemical staining for tumor p-AKT and AKT protein expressions (×200, ×400, ×1000). (B) Immunohistochemical staining for tumor p-GSK3β and GSK3β protein expressions (×200, ×400, ×1000). (C) Immunohistochemical staining for tumor β-catenin and E-cadherin protein expressions (×200, ×400, ×1000). (D) Immunohistochemical staining for tumor MMP-2 and MMP-9 protein expressions (×200, ×400, ×1000). (E) Quantitative analysis of expression of p-AKT, AKT, p-GSK3β, GSK3β, β-catenin, E-cadherin, MMP-2 and MMP-9 protein. All data represent the mean ± SD (n = 6). †P < 0.01 vs. control (NS). [file 1479-5876-12-57-S1.zip › 5093287231126122_fig4D.jpeg]

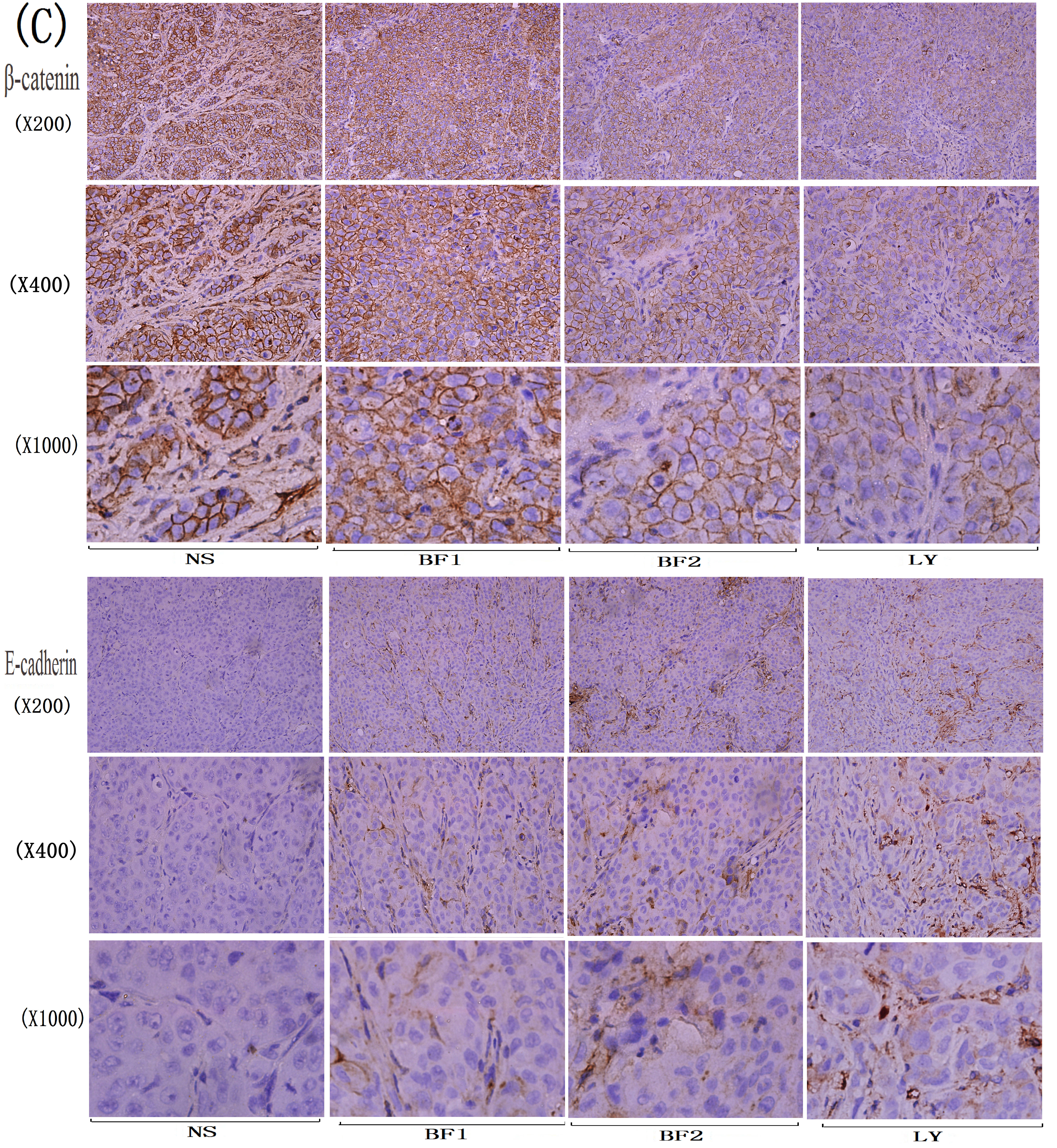

Supplement: Additional file 1: Figure S1 — Bufalin-treated tumors exhibit changes consistent with AKT/GSK3β/β-catenin/E-cadherin signaling pathways in nude mice. (A) Immunohistochemical staining for tumor p-AKT and AKT protein expressions (×200, ×400, ×1000). (B) Immunohistochemical staining for tumor p-GSK3β and GSK3β protein expressions (×200, ×400, ×1000). (C) Immunohistochemical staining for tumor β-catenin and E-cadherin protein expressions (×200, ×400, ×1000). (D) Immunohistochemical staining for tumor MMP-2 and MMP-9 protein expressions (×200, ×400, ×1000). (E) Quantitative analysis of expression of p-AKT, AKT, p-GSK3β, GSK3β, β-catenin, E-cadherin, MMP-2 and MMP-9 protein. All data represent the mean ± SD (n = 6). †P < 0.01 vs. control (NS). [file 1479-5876-12-57-S1.zip › 5093287231126122_fig4C.jpeg]

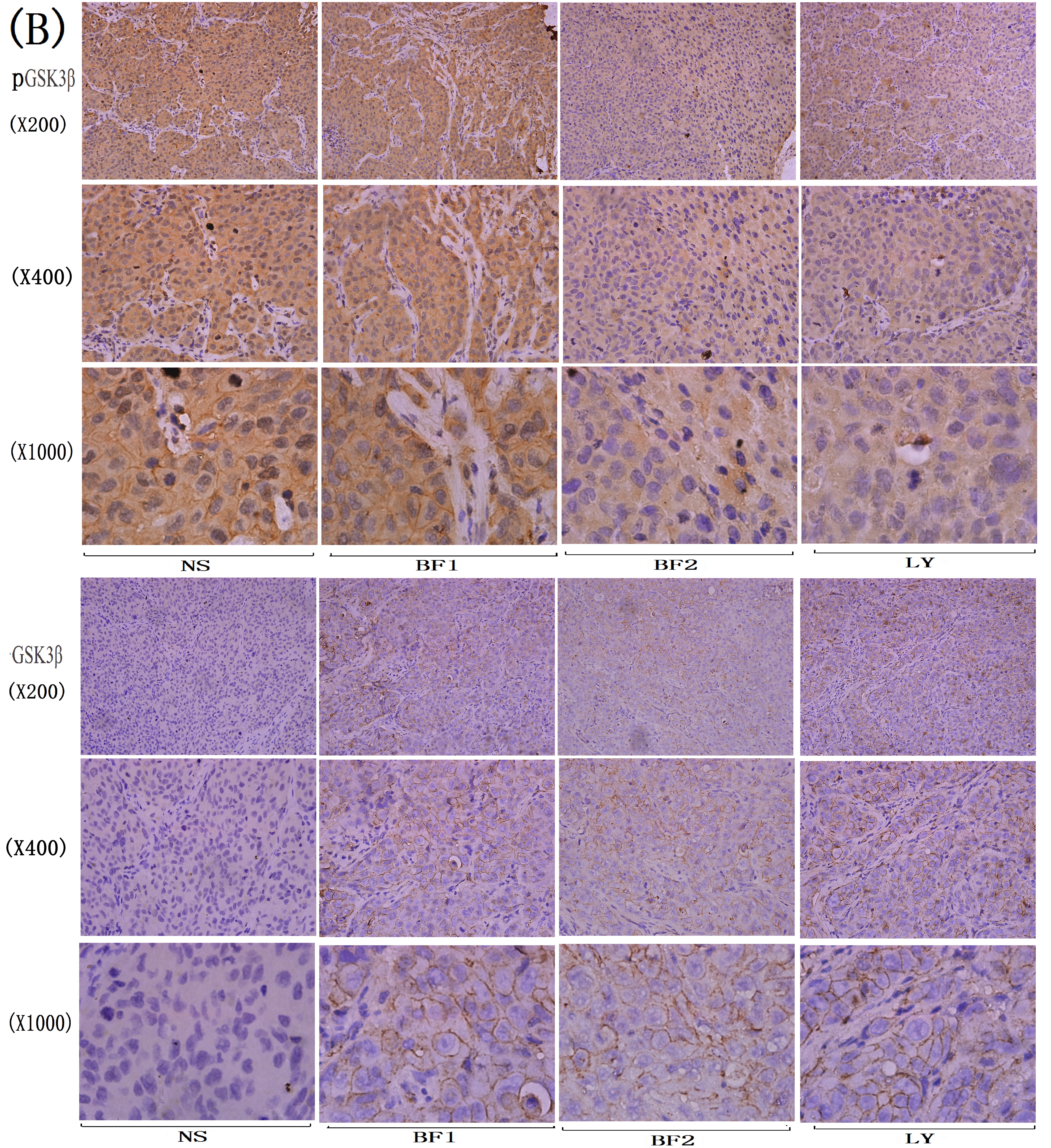

Supplement: Additional file 1: Figure S1 — Bufalin-treated tumors exhibit changes consistent with AKT/GSK3β/β-catenin/E-cadherin signaling pathways in nude mice. (A) Immunohistochemical staining for tumor p-AKT and AKT protein expressions (×200, ×400, ×1000). (B) Immunohistochemical staining for tumor p-GSK3β and GSK3β protein expressions (×200, ×400, ×1000). (C) Immunohistochemical staining for tumor β-catenin and E-cadherin protein expressions (×200, ×400, ×1000). (D) Immunohistochemical staining for tumor MMP-2 and MMP-9 protein expressions (×200, ×400, ×1000). (E) Quantitative analysis of expression of p-AKT, AKT, p-GSK3β, GSK3β, β-catenin, E-cadherin, MMP-2 and MMP-9 protein. All data represent the mean ± SD (n = 6). †P < 0.01 vs. control (NS). [file 1479-5876-12-57-S1.zip › 5093287231126122_fig4B.jpeg]

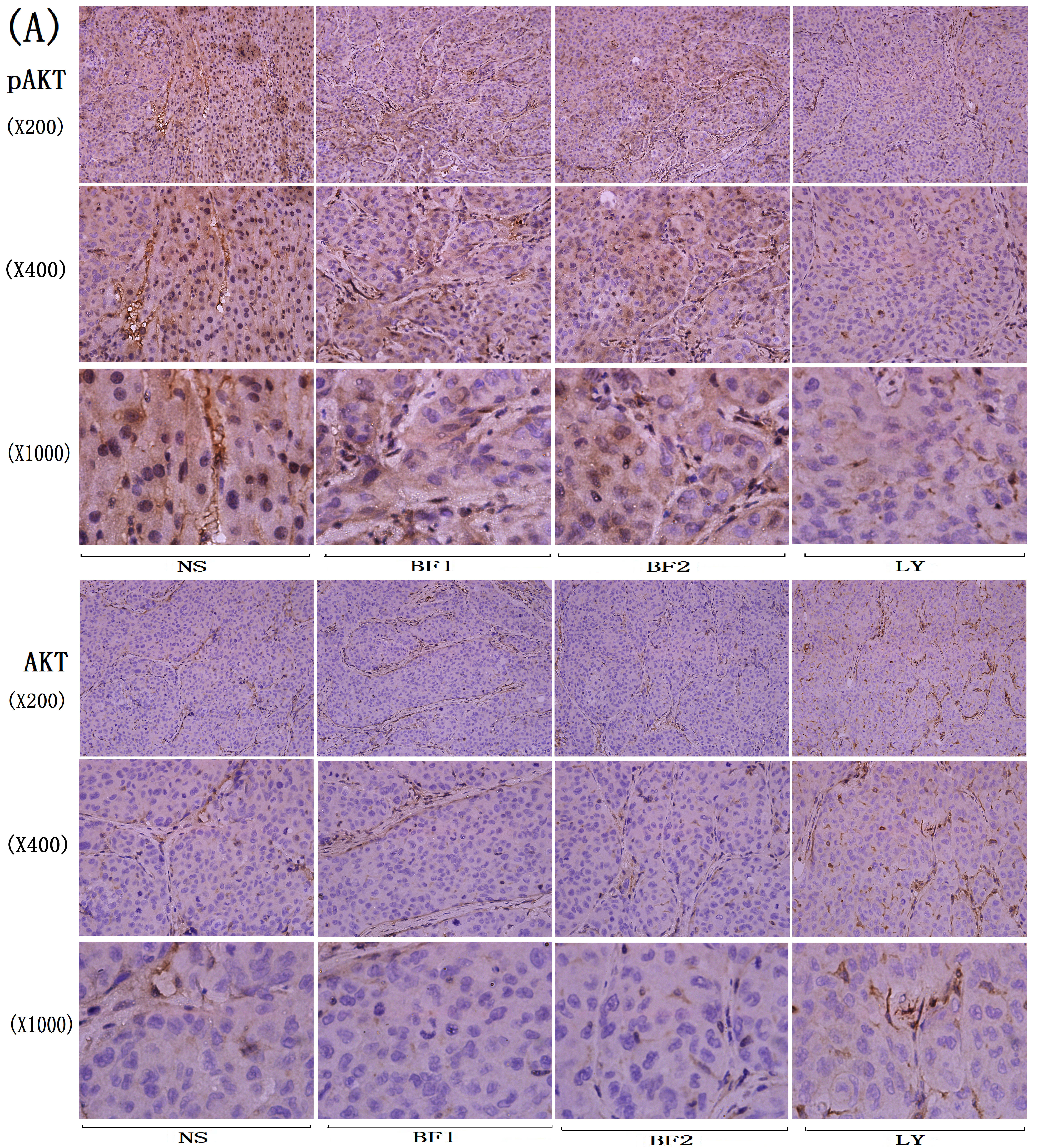

Supplement: Additional file 1: Figure S1 — Bufalin-treated tumors exhibit changes consistent with AKT/GSK3β/β-catenin/E-cadherin signaling pathways in nude mice. (A) Immunohistochemical staining for tumor p-AKT and AKT protein expressions (×200, ×400, ×1000). (B) Immunohistochemical staining for tumor p-GSK3β and GSK3β protein expressions (×200, ×400, ×1000). (C) Immunohistochemical staining for tumor β-catenin and E-cadherin protein expressions (×200, ×400, ×1000). (D) Immunohistochemical staining for tumor MMP-2 and MMP-9 protein expressions (×200, ×400, ×1000). (E) Quantitative analysis of expression of p-AKT, AKT, p-GSK3β, GSK3β, β-catenin, E-cadherin, MMP-2 and MMP-9 protein. All data represent the mean ± SD (n = 6). †P < 0.01 vs. control (NS). [file 1479-5876-12-57-S1.zip › 5093287231126122_fig4A.jpeg]

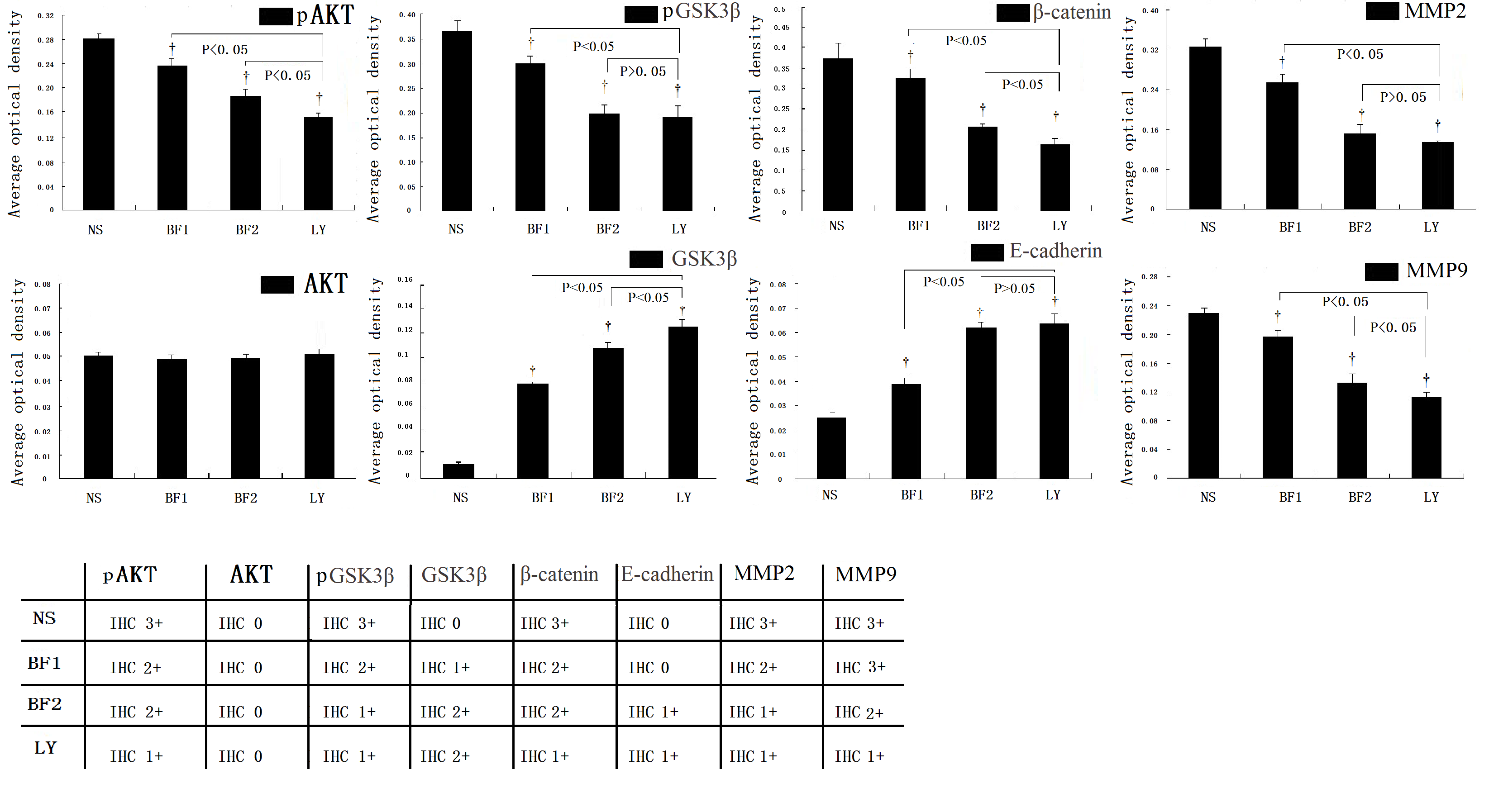

Supplement: Additional file 1: Figure S1 — Bufalin-treated tumors exhibit changes consistent with AKT/GSK3β/β-catenin/E-cadherin signaling pathways in nude mice. (A) Immunohistochemical staining for tumor p-AKT and AKT protein expressions (×200, ×400, ×1000). (B) Immunohistochemical staining for tumor p-GSK3β and GSK3β protein expressions (×200, ×400, ×1000). (C) Immunohistochemical staining for tumor β-catenin and E-cadherin protein expressions (×200, ×400, ×1000). (D) Immunohistochemical staining for tumor MMP-2 and MMP-9 protein expressions (×200, ×400, ×1000). (E) Quantitative analysis of expression of p-AKT, AKT, p-GSK3β, GSK3β, β-catenin, E-cadherin, MMP-2 and MMP-9 protein. All data represent the mean ± SD (n = 6). †P < 0.01 vs. control (NS). [file 1479-5876-12-57-S1.zip › 5093287231126122_fig4E.tiff]
